# Multimodal spatiotemporal transcriptomic resolution of embryonic palate osteogenesis

**DOI:** 10.1038/s41467-023-41349-9

**Published:** 2023-09-14

**Authors:** Jeremie Oliver Piña, Resmi Raju, Daniela M. Roth, Emma Wentworth Winchester, Parna Chattaraj, Fahad Kidwai, Fabio R. Faucz, James Iben, Apratim Mitra, Kiersten Campbell, Gus Fridell, Caroline Esnault, Justin L. Cotney, Ryan K. Dale, Rena N. D’Souza

**Affiliations:** 1 Section on Craniofacial Genetic Disorders, Eunice Kennedy Shriver National Institute of Child Health and Human Development (NICHD), National Institutes of Health (NIH), Bethesda, MD USA; 2 Department of Biomedical Engineering, University of Utah, Salt Lake City, UT USA; 3 School of Dentistry, University of Maryland, Baltimore, MD USA; 4 School of Dentistry, University of Alberta, Edmonton, AB Canada; 5 School of Dental Medicine, University of Connecticut, Farmington, CT USA; 6 Molecular Genomics Core, Eunice Kennedy Shriver National Institute of Child Health and Human Development (NICHD), National Institutes of Health (NIH), Bethesda, MD USA; 7 Bioinformatics and Scientific Programming Core, Eunice Kennedy Shriver National Institute of Child Health and Human Development (NICHD), National Institutes of Health (NIH), Bethesda, MD USA; 8 Department of Genetics and Genome Sciences, University of Connecticut, Farmington, CT USA

**Keywords:** Bone development, Differentiation, Embryology

## Abstract

The terminal differentiation of osteoblasts and subsequent formation of bone marks an important phase in palate development that leads to the separation of the oral and nasal cavities. While the morphogenetic events preceding palatal osteogenesis are well explored, major gaps remain in our understanding of the molecular mechanisms driving the formation of this bony union of the fusing palate. Through bulk, single-nucleus, and spatially resolved RNA-sequencing analyses of the developing secondary palate, we identify a shift in transcriptional programming between embryonic days 14.5 and 15.5 pinpointing the onset of osteogenesis. We define spatially restricted expression patterns of key osteogenic marker genes that are differentially expressed between these developmental timepoints. Finally, we identify genes in the palate highly expressed by palate nasal epithelial cells, also enriched within palatal osteogenic mesenchymal cells. This investigation provides a relevant framework to advance palate-specific diagnostic and therapeutic biomarker discovery.

## Abstract (web summary)

Multimodal spatiotemporal transcriptomic resolution of palatal osteogenesis identifies previously unreported enriched genes in developing palate, paving the way toward viable diagnostic and therapeutic targets for cleft palate disorders.

## Introduction

The dynamic morphogenetic processes required for complete development of the palate are often disrupted by genetic and/or environmental insults^1–4^. Palatal clefts (a sub-set of orofacial cleft anomalies) together with cleft lip are among the most common birth anomalies in humans, occurring in approximately 1 in 700 live births^5,6^. Such birth anomalies pose significant physical, mental, psychosocial, and financial burden on patients and their caregivers throughout life, often requiring multiple stages of complex surgeries with varying rates of success^7,8^. The pathophysiology of palatal clefts is complex and is proposed to involve disturbances in cell proliferation^9^, migration^10^, epithelial-to-mesenchymal transformation (EMT)^11^, cell–cell adhesion^12^, or terminal osteogenic differentiation^13^, which ultimately lead to the failure of palatal shelf fusion^6^. It is known that osteogenesis is a key stage of palatal shelf medial-growth and secondary palate fusion, the failure of which means a viable bony bridge separating oral and nasal cavities fails to form, known as a submucosal cleft palate^14^. While palatogenesis has been studied and characterized morphologically, the molecular mechanisms driving final palatal fusion and osteogenesis remain elusive^15,16^. If properly explored, such information could be applied to the development of pre-clinical models to trial therapeutics that may benefit individuals with submucosal cleft palate^17^ who currently face complex surgeries and life-long care^7^.

Therefore, we identified the marker genes which are likely driving secondary palate fusion. Specifically, we observed the morphogenetic gradients of expression of key osteogenic cues orchestrating the patterning of the secondary palate through time and space. Our studies sought to contextualize gene expression patterns via multimodal data to assess palatal osteogenic patterning and differentiation, offering a first-of-its-kind spatiotemporal resolution of transcriptomic profiles of the developing secondary palate.

## Results

### A stage-specific palate transcriptomic shift identified

**Fig. 1:**
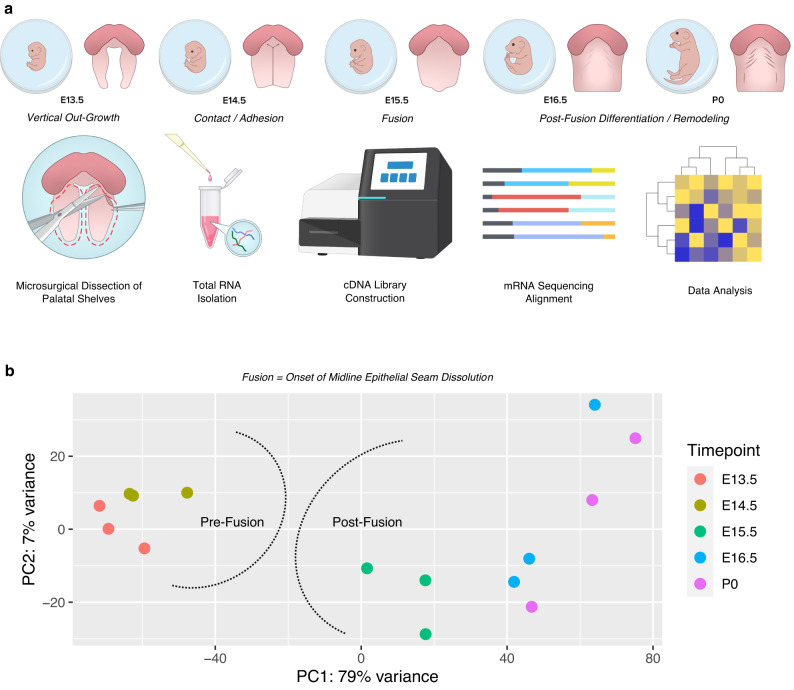
Global transcriptomic profiling of the embryonic secondary palate reveals unique signatures pre- and post-fusion. **a** Total RNA was isolated from microdissected palatal shelves from embryonic stages E13.5, E14.5, E15.5, E16.5, and P0 (*n* = 3 pooled biological replicates and *n* = 3 technical replicates per developmental stage), then run through bulk RNA-sequencing. **b** Principal component analysis (PCA) of all replicates demonstrating global associations in earlier development (pre-fusion) vs. later development (post-fusion) of the secondary palate.

First, we profiled whole-transcriptome gene expression signatures across several stages of palatogenesis (E13.5, E14.5, E15.5, E16.5, and P0) (Fig. 1a). Transcriptomic signatures were broadly grouped into pre-fusion (E13.5–E14.5) and post-fusion (E15.5–P0) categories, as we defined the fusion event as the onset of midline epithelial seam dissolution (between E14.5 and E15.5^15^; Fig. 1b). Our data suggest that a transcriptomic shift exists at palatal fusion. Through differential comparisons of adjacent stages of development (E13.5 vs. E14.5; E14.5 vs. E15.5; E15.5 vs. E16.5; E16.5 vs. P0), we determined that the stage of palatal fusion (E14.5 vs. E15.5) demonstrates the greatest overall shift in gene expression based on *Z*-score (gene abundance) profiling and differential expression (Supplementary Fig. 1).

### Palatal osteogenesis begins with fusion

**Fig. 2:**
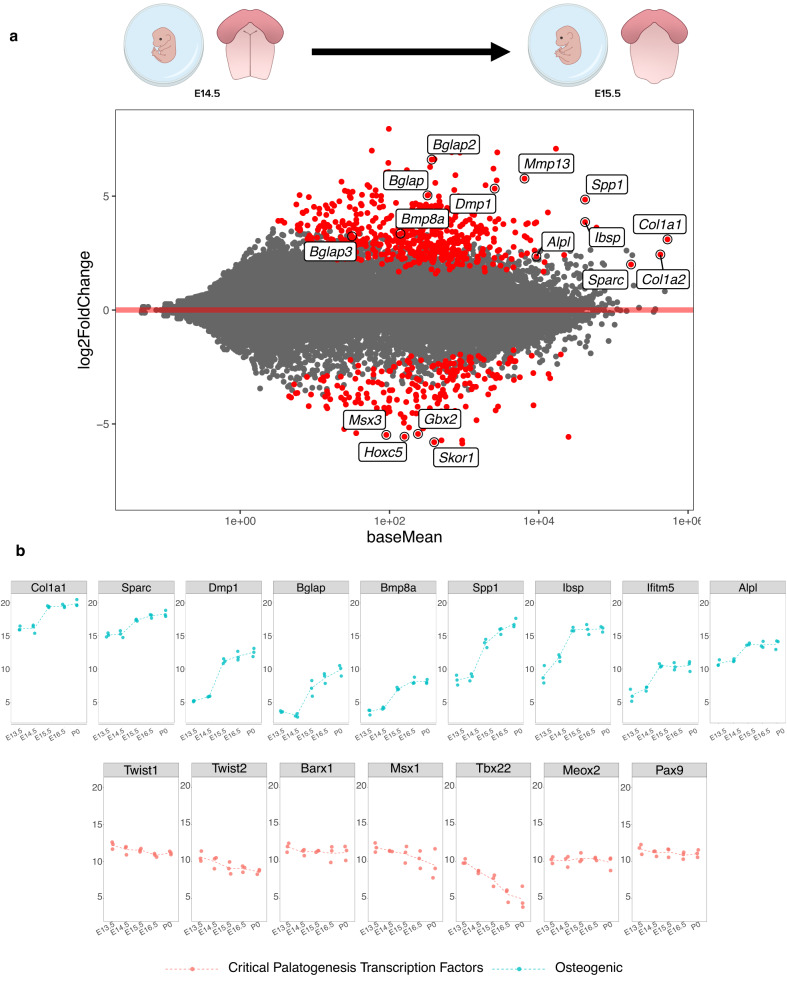
Differential shift toward osteoblast commitment defines the transition from E14.5 to E15.5 in palate development. **a** M–A plot highlights statistically significant (red) differentially expressed genes identified in transcriptomic comparison of E14.5 to E15.5 in development. Genes related to osteoblast lineage commitment and differentiation (up-regulated: *Bglap/2/3, Col1a1, Col1a2, Dmp1*, Spp*1, Alpl, Sparc, Mmp13, Bmp8a;* down-regulated*: Msx3, Gbx2, Hoxc5, Skor1*) were only shown to be differentially expressed at this transitory stage of palatal fusion. **b** Plotting individual genes across all time points studied demonstrated the noted shift in relative osteogenic gene abundance from E14.5 to E15.5, while the same shift was not seen across known critical transcription factors involved in palatogenesis, such as Twist1/2, Barx1, Msx1, Tbx22, Meox1, and Pax9. All bulk RNA-seq analyses derived from *n* = 3 biological and *n* = 3 technical replicates per developmental stage.

Through differential expression analysis of the palatal fusion timepoints, we noted the onset of osteogenic programming at E15.5, supported by numerous significantly enriched genes that are known to either drive or mark osteoblast commitment and differentiation (*Bglap/2/3*^18^*, Mmp13*^19^*, Dmp1*^20^, Spp*1*^21^*, Ibsp*^22^*, Bmp8a*^23^*, and Alpl*^24^) as well as those that mark the formation of critical structural components of bone (*Col1a1*^25^*, Col1a2*^26^*, Sparc*^27^). We also observed down-regulated expression of negative regulators of Msx1 activity (*Msx3*^28^), Wnt signaling (*Skor1*^29^), neural crest migration (*Gbx2*^30^), and skeletal matrix differentiation (*Hoxc5*^31^) at the same stage (Fig. 2a). This onset of osteogenic programming was corroborated by gene ontology enrichment analysis on differentially expressed genes enriched at the E15.5 stage of palatal fusion, highlighting genes and pathways associated with osteoblast differentiation and function, as well as the emergence of genes associated with primary cilia function (Supplementary Fig. 2). Notably, gene markers indicative of osteoblast commitment and differentiation universally increased in expression *only* from E14.5–E15.5, but not across other timepoints. Also, expression profiles of key up-stream regulatory transcription factors known to orchestrate palatogenesis, including *Twist1*, *Twist2*, *Barx1*, *Msx1*, *Tbx22*, *Meox2*, and *Pax9* (Fig. 2b) further support the osteogenic switch in transcriptomic signature occurring at this stage. These global transcriptomic signatures indicating this shift in osteogenic programming from E14.5 to E15.5 led us to focus on these two stages of development.

### snRNA-seq validates osteogenic gene expression at E15

**Fig. 3:**
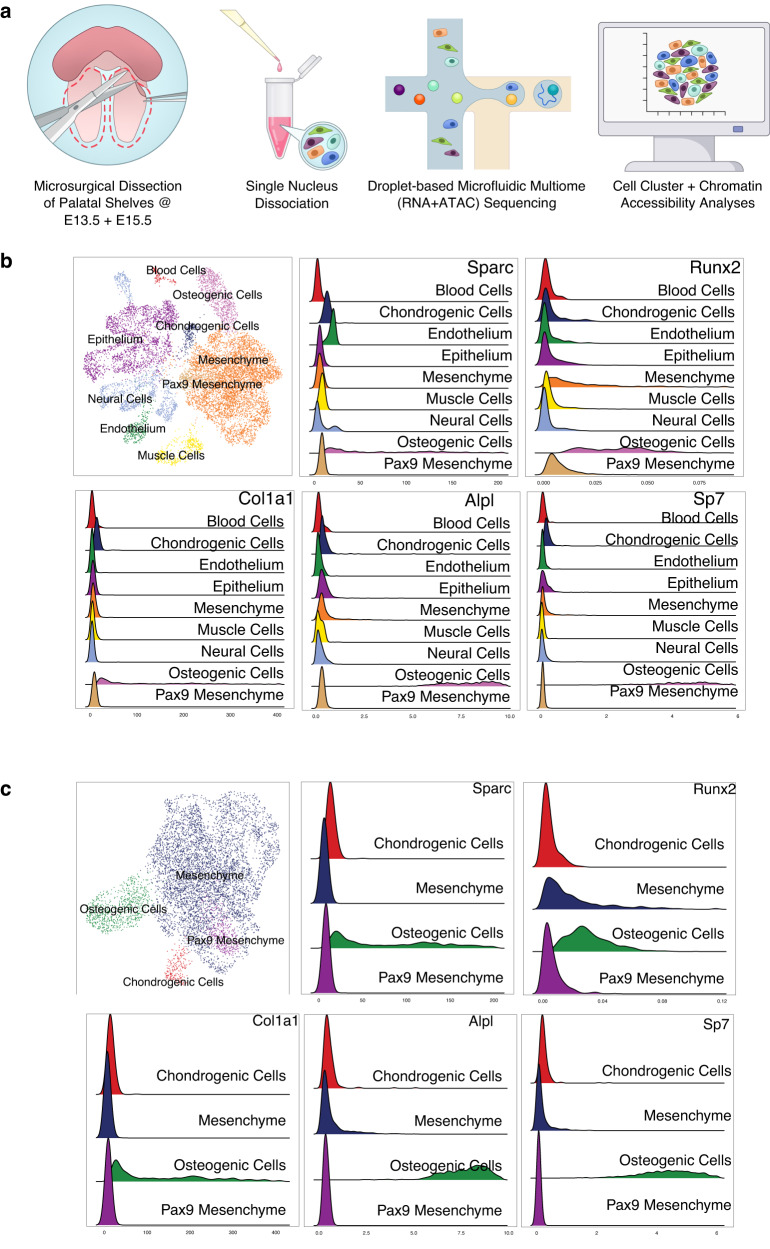
Single-nucleus RNA-seq validation of osteogenic transcriptional programming at E15.5. **a** Microsurgical dissection of age-matched littermates’ (pooled *n* = 3 biological triplicates) secondary palatal shelves in E13.5 and E15.5 mouse embryos was performed to isolate single nuclei suspensions for droplet-based Multiome RNA + ATAC-sequencing (10× Genomics). **b** Validation of bone marker expression profiles specifically within E15.5 secondary palate samples. **c** Further sub-clustering of mesenchyme-specific cell populations.

To further dissect and validate the transcriptomic shift identified in our bulk RNA-seq dataset, we investigated cell type-specific gene expression and binding motif enrichment via integrated single-nucleus gene (snRNA)- and transposase-accessible chromatin (snATAC)-sequencing of microdissected secondary palate tissues from E13.5 and E15.5 mouse embryos. Initial clustering of this dataset revealed ten distinct cell states (Supplementary Fig. 3). Gene ontology enrichment analysis of marker genes from these populations determined the presence of six general cell types: epithelium, mesenchyme, muscle cells, neural cells, endothelium, and blood cells. Further isolated analysis of the mesenchyme identified eight subtypes of this population. Canonical marker genes separate these cells into four broad categories: osteogenic cells, chondrogenic cells, generalized mesenchyme, and *Pax9+* mesenchyme (Supplementary Fig. 4). Interrogation of canonical osteogenic marker genes supported our initial annotation of osteogenic cells present at the E15.5 timepoint, which were absent at the E13.5 timepoint (Fig. 3). This biased presence of osteogenic marker expression to this later timepoint supports our finding of the osteogenic transcriptional programming enrichment by the E15.5 stage.

### In vivo validation and spatial mapping of osteoblast markers

**Fig. 4:**
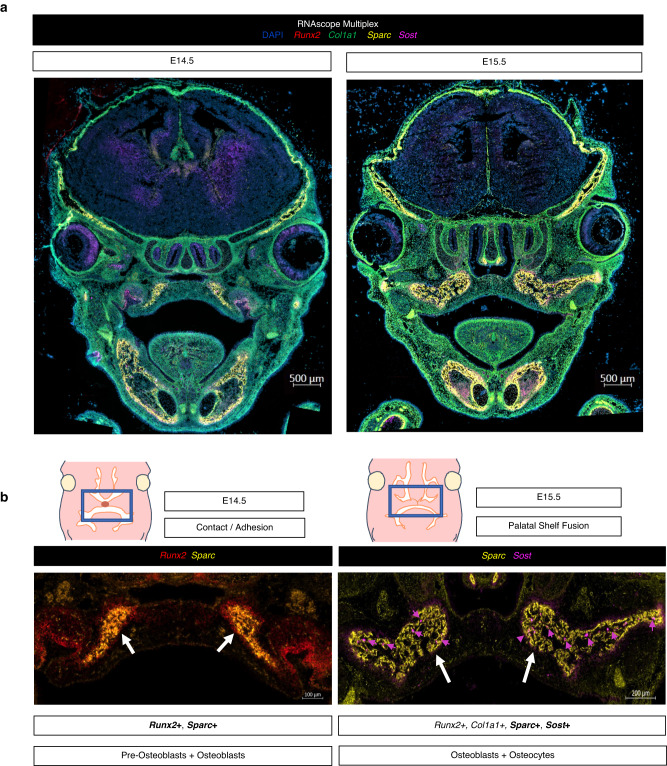
RNAscope Multiplex for in vivo validation of spatiotemporal osteoblast marker mRNA expression in secondary palate tissues. **a** Select osteogenic marker genes hybridized to track lineage-specific spatial patterns of differentiation: *Runx2* (pre-osteoblast), *Col1a1* (early functional osteoblast), *Sparc* (mature functional osteoblast), and *Sost* (osteocyte), in mid-palatal coronal sections via RNAscope Multiplex (*All in vivo validation experiments performed in biological and technical triplicate). **b** Proposed spatiotemporal osteogenic cell lineage activation sequence in developing secondary palate mesenchyme. *White arrows denote central ossification centers; Purple arrows indicate *Sost*+ cells identified in E15.5 secondary palate. All in situ experiments were performed in biological triplicate.

With these findings, we looked to confirm our hypothesis that osteogenesis in the palate occurs between E14.5 and E15.5 while retaining structural and morphological context. We employed multiplexed in situ mRNA hybridization (RNAscope) of select marker genes (*Runx2*, *Col1a1*, *Sparc*, *Sost*). Importantly, we sought to co-localize across space and time marker gene transcripts indicative of (1) regulatory activation of osteogenic differentiation (*Runx2*, *Sost*) and (2) structural components of newly forming bone tissue (*Col1a1*, *Sparc*) in situ, noting the spatiotemporal interplay between these stages of osteogenic programming. *Runx2* is known as a reliable marker for pre-osteoblasts^32^, *Sparc* for functional/mature osteoblasts (required for successful osteoblast mineralization of osteoid)^33,34^, *Col1a1* for functional/active osteoblasts^35,36^ (while not exclusive to osteoblasts, it is closely associated in its genetic interaction to *Runx2* and *Sparc* per String DB [Supplementary Fig. 5]), and *Sost* for Wnt-regulatory osteocyte specificity^37,38^. This allowed for in vivo resolution of osteogenic cell relationships identified from transcriptomic data as regulatory mechanisms of osteogenic induction in turn fuel the transcriptional programming for structural components of bone morphogenesis (Fig. 4a). Comparative mid-palatal formalin fixed paraffin embedded (FFPE) coronal sections from E14.5 and E15.5 embryonic stages were selected based on craniofacial complex anatomical landmarks (bulbous nasal septum, tongue attachment). We observed spatiotemporal expression patterns corroborating the sequencing studies, with notable increase in expression density in palatal shelf ossification centers by E15.5. The expression of the pre-osteoblast marker, *Runx2*, as well as *Sparc*, was localized throughout the developing palatal mesenchyme, with noted diffuse signal in central ossification centers (white arrows in Fig. 4b). In contrast, the highly specific osteocyte marker, *Sost*, localized with much lower signal density at E14.5—in line with our bulk RNA-seq findings—followed by a notable increase by E15.5 following palatal fusion (Fig. 4b).

### In situ spatial RNA-seq of palatal fusion

**Fig. 5:**
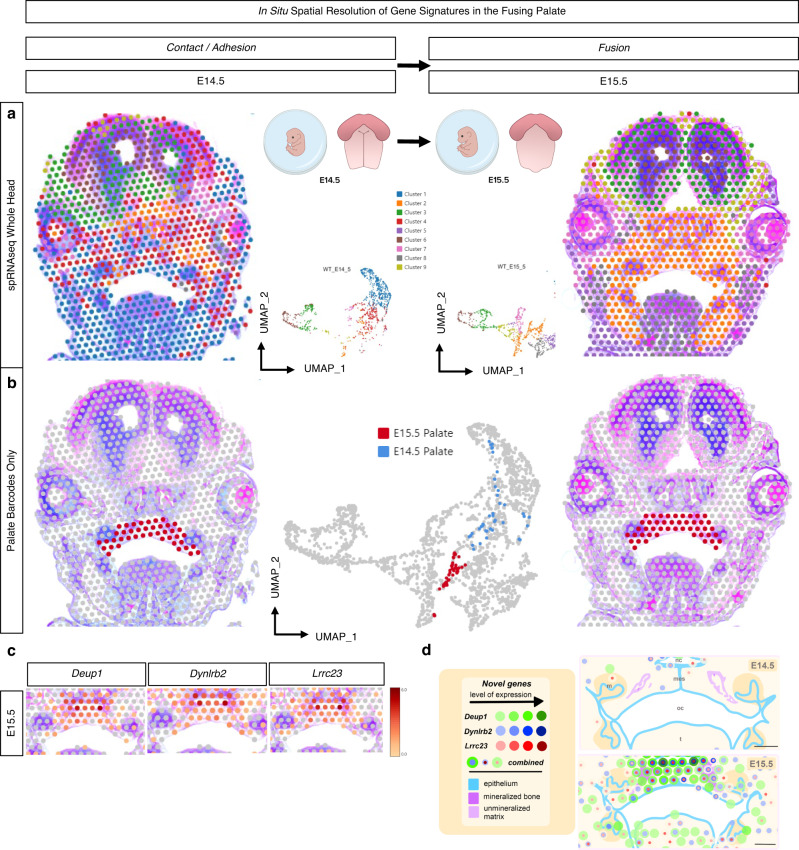
Spatial RNA sequencing (spRNA-seq) for the in situ resolution of the fusing palate’s transcriptome. Mid-palatal coronal cross sections of whole embryo heads were placed on barcoded Visium slide. **a** In vivo clusters were defined from the whole embryo head, demonstrating spatial relationships and morphogenetic diversity of expression, further filtered for only those clusters encoded on the barcodes placed within the palate tissue in each respective section to identify top differentially expressed genes (DEG’s) **b** from E14.5 vs. E15.5 in the palate. *Denotes previously unreported palate-enriched genes identified in spRNA-seq. **c** Spatial gene expression feature plots for the three enriched genes identified. **d** Schematic summary. Colored circles correspond to 55 µm-diameter Visium transcriptomic resolution. Increased expression levels, delineated using the 10X Genomics Loupe Browser, are represented here with darker shades of green (*Deup1*), blue (*Dynlrb2*), or red (*Lrrc23*). The combined localization of these genes is indicated by overlapping concentric colored circles, the diameter of which does not correspond to degree of expression. nc nasal cavity, oc oral cavity, t tongue, mes midline epithelial seam; scale bar: 200 µm. All spatial RNA-seq experiments were performed in biological triplicate.

While bulk and single-nucleus RNA-seq approaches to study the developing secondary palate provide meaningful global temporal transcriptomic data, these approaches lack tissue-specific spatial information critical to inferring genetic regulatory networks in vivo. Although some in vivo validation can occur through the lens of RNAscope, it is primarily a qualitative assay. Further, these in vivo marker validation assays are inherently limited due to multiplexed channel maximums within a narrow spectrum of visibility. Moreover, it is known^39^ that the single-cell suspension, dissociation, and fixation processing steps involved in the 10x Genomics scRNA-seq workflow carry an inherent risk of altering cellular homeostasis. This could lead to physiologically unimportant differential expression observed in cell clusters. We, therefore, employed spatially resolved RNA-seq (spRNA-seq) (Visium, 10x Genomics) on FFPE coronal mid-palatal sections from E14.5 and E15.5 embryos to enable real-time assessment of in situ gene expression and generate a more complete picture of secondary palate fusion (Fig. 5a). While the per-cell resolution is lower than the scRNA-seq workflow, and the 2D nature limits the overall cell capture, the spatial biological insight from sequencing relatively undisturbed tissue slices in situ brings higher confidence in observed spatiotemporal gene expression trends.

Spatial cell cluster heterogeneity was observed globally between distinct morphological zones of the craniofacial complex in coronally oriented mid-cranial sections from E14.5 and E15.5 embryonic stages (Fig. 5a). For example, unsupervised cluster identities of mid-face anatomical structures were more diverse in E14.5 (e.g., clusters 1, 2, 4, and 8), while by E15.5 cluster identities appeared more regionalized and tissue-specific, perhaps indicating a shift in differentiation between these two stages. Barcodes representing the secondary palate in situ tissue arrangement were manually selected within the 10x Genomics Loupe Browser, allowing for a targeted integration of transcriptomic data specific to the palate in each section. Whole-transcriptome differential expression of selected palate tissue barcodes between E14.5 and E15.5 tissue sections (Fig. 5b) highlighted marker genes of potential significance in the processes of secondary palate fusion, including genes related to cell cycle regulation, cilia functionality, cell motility, protein-protein interactions, as well as epithelial and osteogenic cell differentiation (Fig. 5c, d, Table 1). To interrogate the spatiotemporal osteogenic cell lineage differentiation milestones in palatal mesenchymal cells at the time of fusion in situ, known markers of osteogenic progenitors (*Six2, Erg, Bmp7*), pre-osteoblasts (*Runx2*, Sp7, *Mmp13*), osteoblasts (*Ibsp*, *Ifitm5*, and Spp*1*), and osteocytes (*Sost*, *Dmp1*, *Phex*) were identified spatially at each stage (Supplementary Fig. 6a–d). Markers from early osteogenic cell types (osteoprogenitors, pre-osteoblasts, and osteoblasts) demonstrated some expression at E14.5; however, all osteocyte conserved markers only appeared in substantial expression by E15.5—remaining spatially restricted to the mineralized bone (lateral), with some condensed expression noted within the midline site of fusion (mesial), perhaps suggesting multiple spatially distinct regions of osteogenic differentiation within the fusing palate mesenchyme.

**Table 1:** Differential expression analysis of E14.5–E15.5 secondary palate spatial RNA-seq (spRNA-seq) reveals previously unreported (*) genes enriched in expression in palate epithelium and mesenchyme at E15.5

Top 5 DEG’s from E14.5 vs. E15.5 spRNA-seq		
Gene name	Log2Fold change	Gene function
*Cdc20b*	4.436	Cell cycle regulator
*Deup1	4.049	Epithelial cell differentiation
*Dynlrb2	3.612	Microtubule-based cell motility
*Lrrc23	3.415	Protein–protein interactions
*Tnn* (*Tnw*)	3.232	Osteogenic differentiation

### Spatial characterization of fusing palatal shelves in situ

With an eye toward discovery, we turned our attention to the most highly differentially expressed genes identified from the spRNA-seq analysis to identify potential markers of palatal fusion across space and time. By selecting only the palate tissue area on the Visium slide, we directly compared spatiotemporal palate tissue-level gene expression changes. The top five DEGs identified through spRNA-seq of palatal fusion were then compared to our previously generated bulk RNA-seq dataset to corroborate the temporal evolution of expression levels through all embryonic time points studied (Supplementary Fig. 7), which affirmed the differential up-regulation in expression from E14.5 to E15.5. This suggests that these genes may in fact be notable targets for further investigation.

### Spatial resolution of osteoblast maturation at palate fusion

**Fig. 6:**
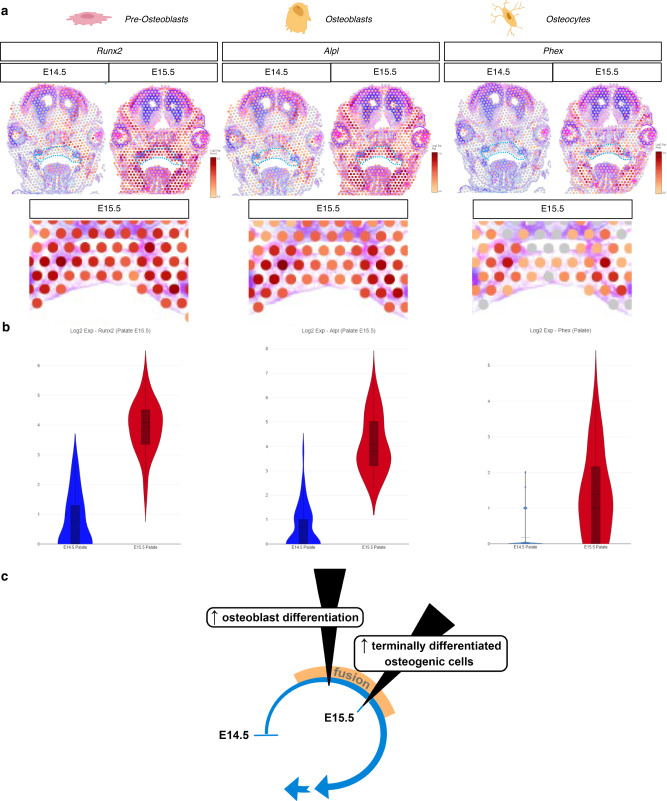
Multimodal transcriptomics resolves spatiotemporal progression of palatal osteogenic cell differentiation. **a** Spatial resolution of select osteogenic differentiation markers delineates cell maturation state from E14.5 to E15.5 in the palate. **b** Violin plots from spatial barcodes quantify relative expression of each gene marker at each respective stage of development, including Log2Fold expression of *Runx2* (E14.5, max: 2.807, q3: 1, mean: 0.699; E15.5, max: 5.672, q3: 4.459, mean: 4.044), *Alpl* (E14.5, max: 3.807, q3: 1, mean: 0.693; E15.5, max: 6.794, q3: 5.107, mean: 4.140); and *Phex* (E14.5, max: 2, mean: 0.176; E15.5, max: 4.248, q3: 2.161, mean: 1.383). **c** Schematic summary of osteogenic differentiation timeline based on transcriptomic signatures identified in our multimodal transcriptomic and epigenetic analyses. *Blue dotted selection represents region of palate barcode selection (as shown in Fig. 5b) for spatial differential gene expression analysis. All spatial RNA-seq experiments were performed in biological triplicate.

To assess these multiple whole-transcriptome assays in parallel, we took a cross-section through the bulk and spRNA-seq datasets with specific osteogenic cell type markers, *Runx2* (pre-osteoblast)^40^, *Alpl* (osteoblast)^41^, and *Phex* (osteocyte)^42^. Osteoblast lineage differentiation significantly increases within the secondary palate mesenchyme from E14.5 to E15.5 (Fig. 6a–c). Osteocytes were found to be spatially restricted in expression lateral to less differentiated osteoblasts, which in turn were more enriched in expression to pre-osteoblast gene markers, identified medially toward the mid-palatal suture, noting condensed expression enrichment of these early markers of osteogenic commitment within the midline mesenchyme of the newly fused palate (Fig. 6b). This suggests that by E15.5 intramembranous ossification of the secondary palate mesenchymal primordia is actively commencing, but most of these mesenchymal osteogenic lineage-committed cells have not yet reached terminal differentiation (osteocyte) (Fig. 6c). Osteocytes likely become more functionally active in the palate mesenchyme during later stages of development following mineralization and maturation of bone matrix tissues^43,44^.

### Cilia-associated enriched genes co-localize with osteoblasts

**Fig. 7:**
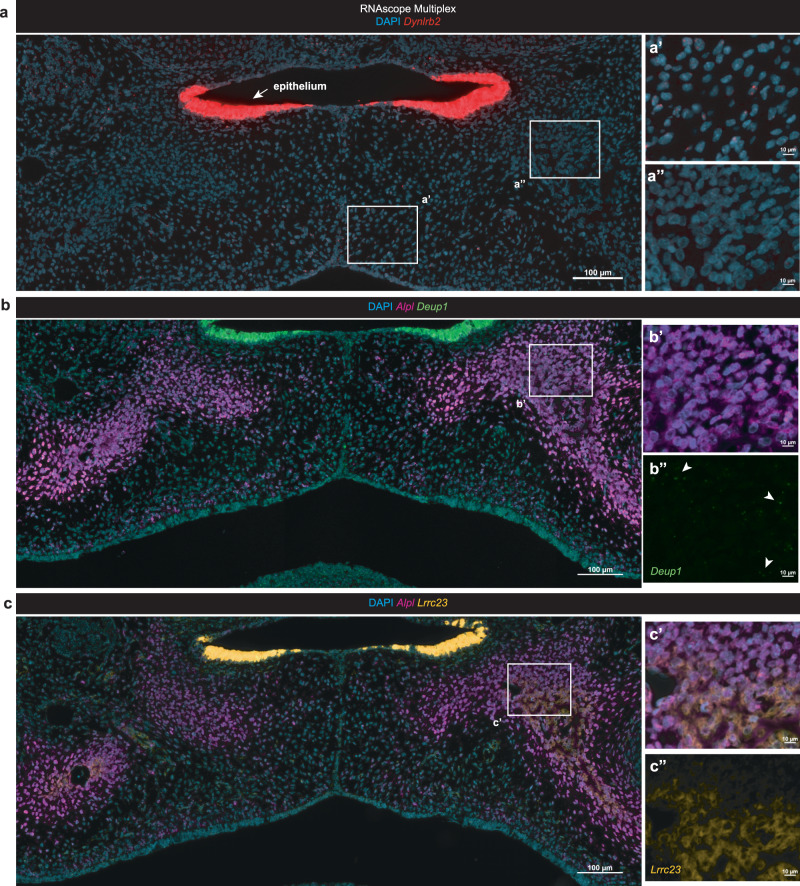
Palate-enriched genes, *Lrrc23* and *Deup1*, co-localize in expression profile at palate fusion with osteogenic cells. RNAscope Multiplex in E15.5 coronal mid-secondary palate of enriched genes *Dynlrb2* (red), *Deup1* (green), *Lrrc23* (gold) identified in spatial RNA-seq co-localized with the osteodifferentiation marker *Alpl* (magenta). **a** *Dynlrb2* expression was highest in the nasal epithelium, with comparatively lower mesenchymal expression in the medial aspect of the palatal shelves (**a’**), but not in the forming bone (**a”**). **b** *Deup1* expression localized to nasal epithelium, as well as palatal osteogenic mesenchyme, expressed by *Alpl*+ osteogenic cells (**b’–b”**). **c** *Lrrc23* was highly expressed by nasal epithelium, with the faint but notable signal of expression within *Alpl*+ palatal osteogenic mesenchyme (**c’–c”**). All in situ experiments were performed in biological triplicate.

Finally, we hypothesized that, given the temporal and spatial alignment of transcriptomic shifts in osteoblast differentiation markers alongside the enriched genes (*Dynlrb2, Deup1, and Lrrc23*), there may be a functional role for these molecules in the osteodifferentiation process occurring during palate fusion. At single-molecule mRNA transcript resolution, we investigated the co-expression profiles of these three genes in-tandem with a marker of osteoblast differentiation, *Alpl* (Fig. 7). We found that at the stage of palate fusion (E15.5), all three of these genes localized intensely to the ciliated nasal epithelium (Fig. 7a–c). This was not surprising, given their known roles in primary cilia function. More surprisingly, all three of these genes did demonstrate fluorescent signal at single molecule resolution within the palate mesenchyme, as well. Specifically, *Dynlrb2* expression was noted to be highest in the nasal epithelium, demonstrating comparatively lower expression within the mesial palate mesenchyme (Fig. 7a’), but not within the forming bone (Fig. 7a”). *Deup1* expression localized to the nasal epithelium and, intriguingly, also was co-expressed by cells in the palate osteogenic mesenchyme expressing *Alpl* (Fig. 7b–b”), potentially implicating a functional role for *Deup1* in osteodifferentiation during palatal fusion. *Lrrc23* transcript localization was enriched with notable signal within *Alpl+* palatal osteogenic mesenchyme (Fig. 7c–c”) in addition to its intense signal demarcating the nasal epithelium.

## Discussion

In our analyses, a transitory phase was identified between E14.5 (defined previously as the stage of contact/adhesion of palatal shelves^15,45^) and E15.5 (the stage of fusion of palatal shelves^15,45^) wherein osteogenic marker genes were identified in higher abundance. Specifically, E15.5 was marked by increased osteoblast differentiation and commitment gene abundance, as well as the introduction of genes identifying terminally differentiated bone cells (osteocytes) within the hard palate mesenchyme. Osteocytes were found to be spatially restricted in expression lateral to osteoblasts and pre-osteoblasts, which had already begun traversing medially toward the mid-palatal suture from their lateral ossification zone of origin, as well as condensed expression enrichment within the midline mesenchyme of the newly fused palate. Importantly, the timeline of transcription for important osteogenic genes identified here provides valuable molecular insights to corroborate what is known histologically through palatal development. Explicitly, the presence of abundant osteogenic gene transcripts at E15.5 warrants further studies on the origin of osteogenic cells spatially and temporally through lineage tracing and live cell imaging modalities as post-fusion development of the mid-palatal suture proceeds.

An important component of this transcriptomic profiling analysis included the identification of up-regulated marker genes not previously reported in the process of palatal fusion (*Deup1*, *Dynlrb2*, *Lrrc23*). These genes were found to be primarily enriched in expression within the palate nasal epithelium at E15.5, likely indicating the active role these specialized cells in the palate play in facilitating cell migration upon fusion of the palatal shelves. This concept of epithelial involvement in palatal fusion processes has been studied in-depth by other groups^10^. Intriguingly, two of these genes (*Deup1* and *Lrrc23*), upon single molecule in situ resolution, were found to co-localize within *Alpl*+ palatal osteogenic cells within the mesenchyme at the stage of palatal fusion, while the third (*Dynlrb2*) was also found expressed in other palatal mesenchymal cells toward the midline, which may point to a role in mesenchymal progenitor cell function. Our approach in utilizing the lower resolution spatially resolved sequencing modality for the first time in the embryonic palate was valuable to identify these three genes highly expressed within palate cell populations. However, the lack of single-molecule sensitivity inherent in this assay implied a gradient of expression beginning from the nasal epithelium and extending into the mesenchyme (Fig. 5d). RNAscope Multiplex in situ hybridization was a necessary and critical extension of these findings to clarify the technical limitation and specific localization at single molecule resolution.

Prior work in palate development has largely focused on known mouse genetic models as the basis of studying genes of relevance to known causative mutations in humans. Now, with the ability to capture entire tissue-specific transcriptomes across space and time, the possibilities for discovery of relevant genes critical for palatogenesis have expanded significantly. *Deup1*, a deuterosome-mediated centrosome amplifier, has a known function of promoting multi-ciliated epithelial cell differentiation^46,47^. Now, our data suggests that its role may extend beyond epithelial cells, as *Deup1*+ palatal osteoblasts reside within condensed mesenchyme of the developing secondary palate. *Lrrc23* is a conserved component of the radial spoke, a structure which facilitates the beating motion of cilia and flagella^48,49^. *Dynlrb2* acts as one of several non-catalytic accessory components of the cytoplasmic dynein 1 complex thought to be involved in linking dynein to cargos and to adapter proteins that regulate dynein function, acting as a motor for the intracellular retrograde motility of vesicles and organelles along microtubules^50^. Notably, global knockout mouse models have been generated previously for each of these three genes, and no phenotype specific to the palate has been reported in the limited analyses performed^46,48–50^. Although, all mutant mice are viable, and two of the three (*Dynlrb2*, *Deup1*) demonstrate growth defects, which may be consistent with an ossification and / or bone growth anomaly. However, these prior reports do not provide sufficient detail to critically examine any potential functional or morphological consequences of genetic deficiency in the craniofacial complex specifically. Furthermore, it is known that patients affected with sub-mucosal cleft palate anomalies may not readily manifest phenotypically upon rudimentary physical exam^51,52^. This can be similarly true in animal models mimicking the disease phenotype. Thus, there remains a need for future studies to better understand at high resolution the direct role cilia-associated molecules such as *Deup1* and *Lrrc23* (both found co-localized to palatal osteogenic cells) may be playing in the propagation of palatal osteogenesis. Given the localization of expression of these two genes within osteogenic cells in the fusing palate, coupled with the understanding of primary cilia’s roles in craniofacial development beyond the palate, further study should investigate the role of specific bone cells (osteoprogenitors, pre-osteoblasts, osteoblasts, and osteocytes) through development and their respective cilia-mediated cell-cell interactions specific to the palate.

It is known that ablation of ciliary structural proteins can lead to inhibited osteoblast differentiation, osteoblast polarity, and an overall reduction in bone formation^53^. The pathway analyses from our bulk RNA-seq dataset, further corroborated in situ, highlight the likely functional importance of primary cilia in orchestrating the differentiation of palatal mesenchyme into palatal bone at (and likely after) the stage of fusion. This provides yet another layer of evidence in support of previous studies which have linked conserved primary cilia function and palatogenesis^54–56^. Taken together, there seems to be a strong biologic rationale in focusing on embryonic palatal osteogenesis to further explore the role of primary cilia in this process, and its potential role in the proper formation of palatal bone.

Given the timeline of osteogenic gene expression observed in our sequencing studies herein, future studies wishing to assess the functional role(s) of osteocytes in differentiating palatal mesenchyme would require studying stages beyond E15.5. This approach is dictated by the markedly low mRNA expression levels of osteocyte-specific markers that were identified in earlier palate developmental stages through bulk, single-nucleus, and spatial RNA-seq modalities. Notably, therapeutic control of osteogenic processes in vivo has been achieved and optimized previously with a targeted Wnt pathway agonist (e.g., Romosozumab, a monoclonal antibody to sclerostin) to modulate the regulatory function of osteocytes for the bolstering of bone tissue^57–59^. The possibility of transiently manipulating the regulatory signaling control exerted on proliferating and differentiating pre- and functional osteoblast cells by osteocytes may provide exciting avenues of potential therapeutic development for congenital deficiencies of craniofacial bone tissues, as our lab^60,61^ and others^62,63^ have demonstrated previously through targeting other up-stream Wnt pathway regulators. As the future of fetal and neonatal medicine includes in-utero preventive and therapeutic interventions, there is a need to continue to decipher the spatiotemporal gene regulatory networks driving normal and abnormal development to then be able to intervene safely and effectively with targeted therapeutics^8,64–67^.

In summary, this study provides for the first time a transcriptome-wide, staged developmental roadmap of temporally and spatially resolved morphogenetic cues culminating in palatal fusion. Enriched genes within the palate were identified and mapped spatiotemporally at the site of fusion, identifying the cilia associated with *Dynlrb2,* *Deup1*, and *Lrrc23* to be highly enriched in the fusing secondary palate nasal epithelium, and more surprisingly, within the mesenchyme, with *Deup1* an *Lrrc23* specifically localized within palate osteogenic cells. Importantly, by providing this dataset to the craniofacial biology community through open-access sharing of all data on FaceBase (the primary shared data source for craniofacial researchers worldwide), we aim to support and facilitate translational studies on palate development. These studies will lead to the development of gene regulatory networks and identification of important nodes for continued discovery, as well as new preclinical models of palate ossification and submucosal clefts, paving the way toward potential therapies to correct cleft palate defects in humans.

## Methods

### Animals

All animal procedures and study protocols were approved by the National Institutes of Health, National Institute of Child Health and Human Development Animal Care and Use Committee (ACUC), under Animal Study Protocol (ASP) #21-031. C57BL/6J *Mus musculus* were obtained from the Jackson Laboratory. Inbred strains of female C57BL/6 *Mus musculus* were utilized for all experiments. Healthy fertile male *Mus musculus* were mated with the same strain C57BL/6J females. Timed pregnancies were conducted via vaginal plug identification, with day 0.5 indicating the date of identification. All racks within the animal facility are individually ventilated cages (IVC) that hold micro-isolated cages. This style of racks maintains low levels of ammonia, humidity, a balanced/consistent air change and temperature within each cage. Facility staff provide Sani-Chip (Envigo T 7090M) as bedding and NIH-07 fixed formula (5018 LABDIET NIH Rat & Mouse Ration NIH-07) as the feed (both are not sterilized). Water provided is municipal tap. Each mouse cage is also provided with one cotton nesting square and 2 g of brown crinkle paper for standard enrichment. The facilities lighting cycle is 14 h (On) 6 a.m.–8 p.m., 10 h (Off) 8 p.m.–6 a.m.

### Palate dissection, single nucleus dissociation

Pregnant *Mus musculus* were sacrificed by CO_2_ inhalation and cervical dislocation. All surgical procedures were performed by using a surgical loupe (Orascoptic, Eye Zoom, ×5.5 magnification). Pregnant *Mus musculus* were placed in the supine position on a sterile, absorbent surgical pad and disinfected with 70% ethanol along the site of planned incision. An incision was made on the abdomen along the midline using small surgical scissors. A fresh microsurgical scissor was then used to carefully incise the peritoneum to expose the uterine chain. Using blunt forceps, the uterine chain was externalized. Embryos were dissected out by releasing it along the myometrium, incising at the oviduct bilaterally and the median uterine horn ligament attachment. Whole embryos were transferred into ice-cold sterile PBS in a 10 cm Petri dish. Each embryo was carefully dissected out from the uterus and extra-embryonic amnion and chorionic tissues then transferred to a new 10 cm culture dish with fresh ice-cold PBS. A blunt forceps was used to hold each embryo and by using a small fine microsurgical scissor, an incision was made on bilateral oral commissures, allowing for extended opening of the mandible and clear vision of the palate cranially. Careful microdissection of the palatal shelves only from respective embryonic stages was performed, with noted potential extra-palatal tissue contamination due to surgical imprecision. Pooled littermates (*n* = 3 biological replicates per sample) of each respective stage were utilized for either total RNA isolation (E13.5, E14.5, E15.5, E16.5, P0) or single-nucleus dissociation (E13.5, E15.5) (10× Genomics).

### Total RNA isolation, bulk RNA-sequencing

Dissected palatal shelves from E13.5, E14.5, E15.5, E16.5, and P0 were used for bulk RNA sequencing. Secondary palate tissue samples from 3 embryos were pooled into 1.5 mL tubes and placed immediately on dry ice and stored in −80 °C. For each stage of analysis, three technical replicates and three biological replicates were included for sequencing. Tissues were homogenized using biomasher II. mRNA was extracted and purified using Macherey Nagel™ NucleoSpin™ mini kit. 1–4 μg of total RNA samples were purified with PolyA extraction, and then purified mRNAs were constructed to RNA-Seq libraries with specific barcodes using illumina TruSeq Stranded mRNA Library Prep Kit. All the RNA-Seq libraries were pooled together and sequenced using illumina NovaSeq to generate approximately ~40 million 2 × 100 paired-end reads for each sample. The raw data were demultiplexed and analyzed further. Raw sequence reads were processed using lcdb-wf v1.9rc (lcdb.github.io/lcdb-wf/) according to the following steps: Raw sequence reads were trimmed with cutadapt v3.4^68^ to remove any adapters while performing light quality trimming with parameters ‘-a AGATCGGAAGAGCACACGTCTGAACTCCAGTCA -A AGATCGGAAGAGCGTCGTGTAGGGAAAGAGTGT -q 20 –minimum-length = 25.’ Sequencing library quality was assessed with fastqc v0.11.9 with default parameters. The presence of common sequencing contaminants was evaluated with fastq_screen v0.14.0 with parameters ‘–subset 100000 –aligner bowtie2.’ Trimmed reads were mapped to the Mus musculus reference genome (GENCODE m18) using HISAT2 v2.2.1^69^. Multimapping reads were filtered using samtools v1.12^70^. Uniquely aligned reads were then counted in genes with the featureCounts program of the subread package v2.0.1 using Mus musculus reference (GENCODE m18) annotations^71^. Differential expression was performed using raw counts provided to DESeq2 v1.34.0^72^ with the following modifications from lfcShrink default parameters: type = “normal” and lfcThreshold=1. A gene was considered differentially expressed if the false discovery rate (FDR) was <0.1 (default for DESeq2 for the statistical test that magnitude of the log2FoldChange is >1 (lfcThreshold = 1)). Patterns of expression profile were computed and plotted using the function degPatterns from DEGreport v1.30.0 (http://lpantano.github.io/DEGreport/). Functional enrichment was performed for GO Biological Process, Cellular Component, and Molecular Function using the ClusterProfiler v 4.2.0 function go.enrich^73^.

### Single-nucleus RNA+ATAC sequencing (multiome-RNA+ATAC-seq, 10x genomics)

Samples were mechanically broken into single-nuclei suspensions following the manufacturer’s instructions for Nuclei Isolation from Complex Tissues for Single Cell Multiome ATAC + Gene Expression Sequencing (10X Genomics—protocol CG000375). After isolation, the nuclei integrity was accessed under a microscope and counted using the CellDrop (Denovix) and acridine orange (AO) and propidium iodide (PI) as dye. Transposition and Gel Bead-in-Emulsion (GEM) generation, using a Chromium Single Cell Multiome ATAC + 3′ Gene Expression following the manufacturer’s instructions (10x Genomics—protocol CG000338 Rev F) were performed, aiming for a total of 10,000 nuclei per sample. In brief, the nuclei were transposed in a bulk solution using Tn5 transposase with sequencing adapters that were pre-loaded. The transposed nuclei were loaded onto a microfluidic chip to create GEMs. The gel beads contained two types of oligos that were used for snRNA-seq and snATAC-seq. The mRNA was reverse transcribed and underwent template switching and transcript extension to produce barcoded cDNA. The snATAC-seq oligo was formed of a partial Illumina primer sequence, a 10X barcode, and a spacer that allowed the barcode to attach to the adapter-tagged DNA fragments. The GEMs were broken using a recovery agent to produce a bulk pool of barcoded molecules. The pre-amplified product was then split into portions available for snRNA-seq and snATAC-seq library construction. P5, i7, and P7 sequences compatible with Illumina bridge amplification were added to the DNA fragments through PCR to produce snATAC-seq libraries. Additional amplification of the cDNA was performed on the snRNA-seq samples before proceeding to fragmentation, end repair, A-tailing, and ligation. A sample dual index PCR was used to add the P5, i5, i7, and P7 sequences to the final snRNA-seq library. Sequencing was performed on the Illumina NovaSeq 6000 at the Molecular Genomics Core (NICHD).

### Single-nucleus RNA+ATAC-seq bioinformatics analysis

Raw fastqs were aligned to mm10 genome build using the standard Cellranger multiomics settings and imported to ArchR (v.1.0.1). Doublets were identified and filtered, and cells were filtered for minimum 4 TSS enrichment, 2500 fragments per cell. Dimensionality reduction was performed using LSI based on the cell-by-fragment matrix and cell-by-gene matrix, and clusters were identified. Peaks were called based on original clustering, discarding reads from promoters (2500 bp ± TSS) and exons. Cluster assignments were confirmed using canonical marker genes based on gene expression, and gene ontology enrichment of marker genes identified using getMarkerFeatures function of ArchR. Statistical significance and strength of enrichments were determined using a *t*-test, grouping cells by cluster. Detailed scripts of analysis can be found on the Cotney Lab GitHub (https://github.com/emmawwinchester/mousepalate).

### Fluorescent multiplex mRNA in situ hybridization (RNAscope)

Mouse embryos were collected in biological triplicates at E13.5, E14.5, and E15.5 and fixed in 10% formalin for 24 h. Samples were then processed to paraffin embedding and were sectioned at 5 µm on a microtome. RNAscope multiplex fluorescent v2 assay (Advanced Cell Diagnostics, 323100) was used for in situ hybridization according to the manufacturer’s instructions with a modified pretreatment custom reagent for antigen retrieval. Positive and negative control probes were employed with assistance from Advanced Cell Diagnostics’ Professional Assay Services to ensure quality and reproducibility of RNA assays in our embryonic mouse FFPE samples. Marker probes from Advanced Cell Diagnostics for *Col1a1* (Cat. #319371), *Sparc* (Cat. #466781-C2), *Runx2* (Cat. #414021-C3), *Sost* (Cat. #410031-C4), *Alpl* (Cat. #441161-C4), *Deup1* (Cat. #805591-C2), *Dynlrb2* (Cat. #1243011-C3), and *Lrrc23* (Cat. #1243001-C3) were used in this study. Representative serial slides were also stained with hematoxylin and eosin (H&E) for histomorphometry context. The fluorescent slides were imaged on an AxioScan.Z1 slide scanner (Zeiss) with Plan-apochromat ×40/0.95 objective in five fluorescent channels (DAPI, FITC, Texas Red, Cy5, Cy7).

### Spatial RNA sequencing (spRNA-seq, 10x Genomics, Visium)

All steps from 10X Genomics’ FFPE Visium Spatial workflow were followed. In brief, spatial RNA sequencing (spRNA-seq) slides are coated with an array of poly-T primers, which encode unique spatial barcodes. These barcodes contain thousands of encoded oligonucleotides within a catchment frame of 6.5 × 6.5 mm. The oligonucleotides are selectively hybridized with the 3’ end of mRNA eluted upon tissue permeabilization, enabling scRNA-seq-like mRNA sequencing with the individual barcode spots replacing the individual suspended cells. Each barcoded spot on the slide is ~55 µm in diameter, which is predicted to capture ~10 cells per spot. FFPE tissue was sectioned directly onto the barcoded slide, H&E stained, and then underwent enzymatic permeabilization, which allowed for mRNA release and subsequent capture by primer-coated slides. These mRNA molecules are visualized through the incorporation of fluorescent nucleotides into the complementary DNA (cDNA) synthesis process. The resolution of unique spatial barcoding in situ allows the matching of RNA abundance with the original spatial location in the tissue section, providing a whole-transcriptome RNA-sequencing with 2-dimensional spatial relation. All differential spatial sequencing analyses were conducted using the 10X Genomics Loupe Browser (Version 6) using the standard manufacturer recommendations and protocol.

### Statistics and reproducibility

All experiments were performed using at least *n* = 3 biological and *n* = 3 technical replicates. Statistical significance and strength of enrichments was determined using *t*-test, grouping cells by cluster. A gene was considered differentially expressed if the false discovery rate (FDR) was <0.1 (default for DESeq2 for the statistical test that magnitude of the log2FoldChange is >1 (lfcThreshold = 1)). No data were excluded from analyses. No statistical method was used to predetermine the sample size.

### Reporting summary

Further information on research design is available in the Nature Portfolio Reporting Summary linked to this article.

### Supplementary information

Supplementary Information

Reporting Summary

## Data Availability

All results and analysis data are available in the main text or the supplementary materials. Additional information and materials are available from the corresponding author upon reasonable request. All sequencing data reported in this paper are deposited in GEO under the SuperSeries GSE205449.

## Code availability

Detailed scripts of single-nucleus RNA-seq analysis can be found on the Cotney Lab GitHub (https://github.com/emmawwinchester/mousepalate) and detailed methodology for bioinformatic analysis in the “Methods” section.
