# Supplementary material for: Multimodal spatiotemporal transcriptomic resolution of embryonic palate osteogenesis
Source: Nat Commun. 2023 Sep 14;14:5687. doi: 10.1038/s41467-023-41349-9 (PMC10502152; doi:10.1038/s41467-023-41349-9)
Supplement: Supplementary file 1 — Supplementary Information [file 41467_2023_41349_MOESM1_ESM.pdf]

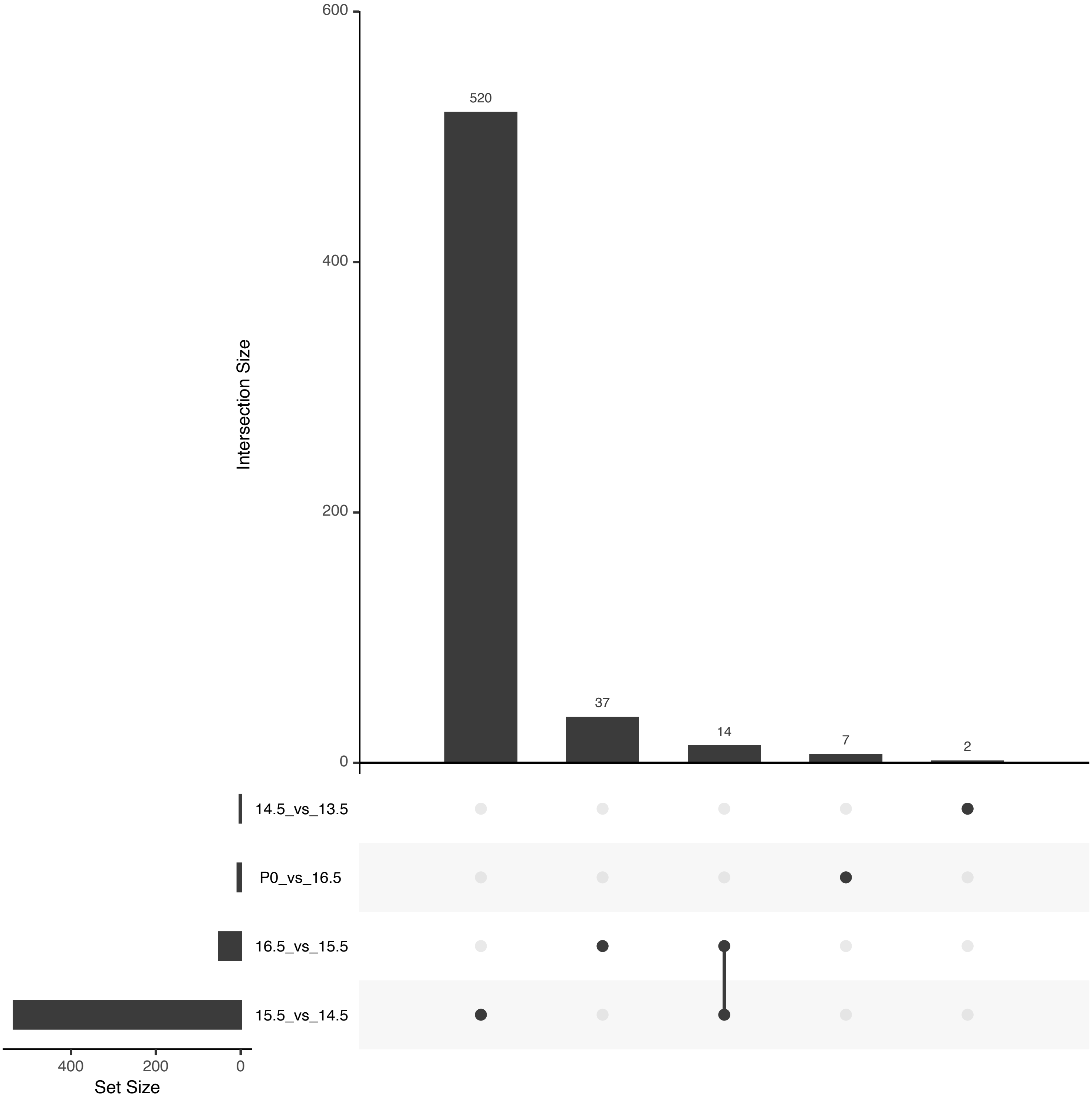

Supp. Fig. 1. Staged differential expression analyses identify E14.5-E15.5 transition as greatest overall shift in gene expression. Up-set plot of all staged comparisons conducted for differential expression analysis (n=3 biological replicates and n=3 technical replicates for each developmental stage analyzed). Closer investigation of the stage of palatal fusion (E14.5-E15.5) demonstrated the greatest shift in overall gene expression compared to all other staged comparisons. *Intersection size indicates number of differentially expressed genes in each staged comparison.

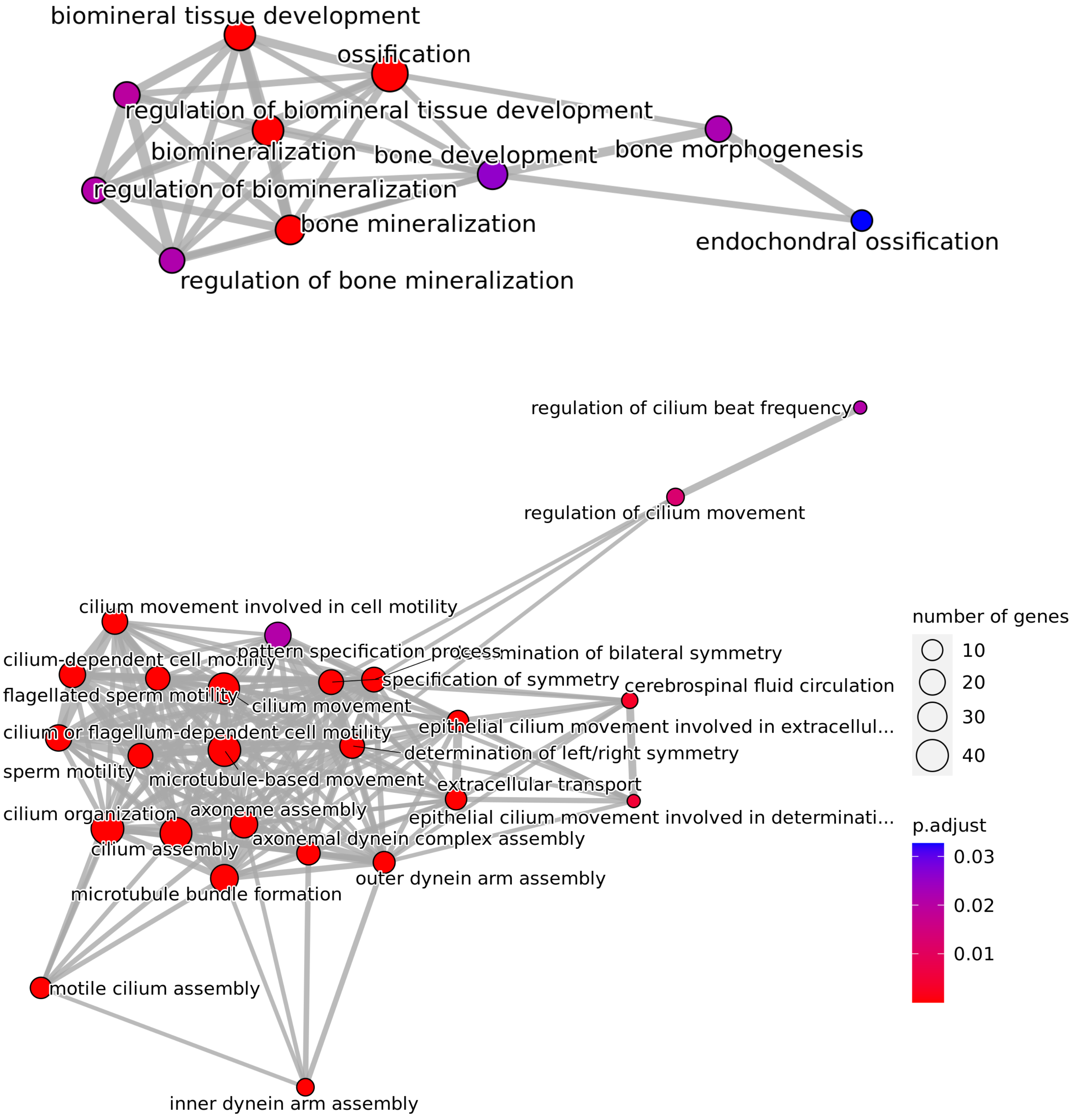

Supp. Fig. 2. Functional Enrichment Analysis from E14.5 vs E15.5 highlights bone morphogenesis and cilia development as key processes at this timepoint. Functional enrichment analyses from GO Biological Processes database corroborated gene patterning associations based on stage-specific signatures identified from single genes.

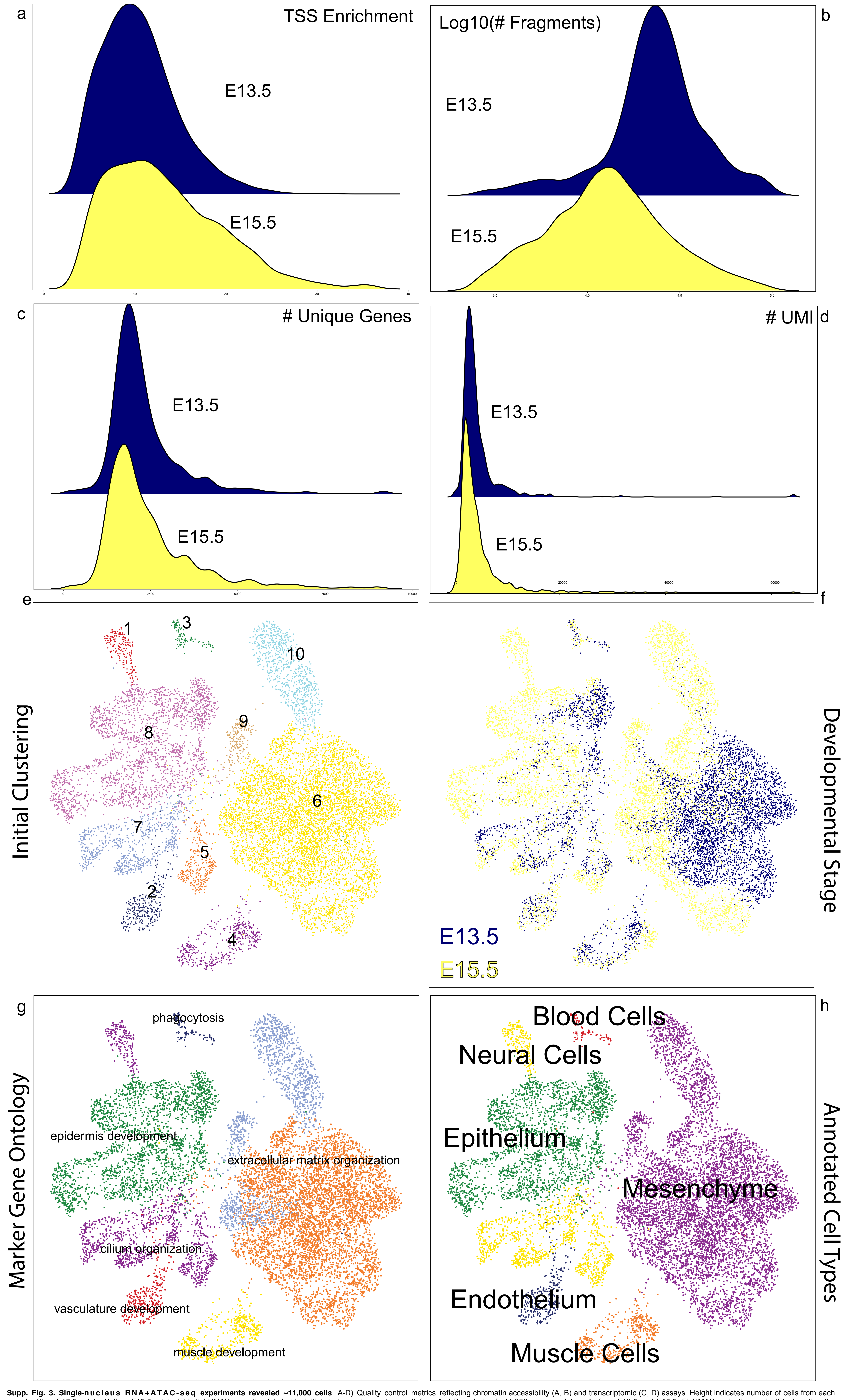

Supp. Fig. 3. Single-nucleus RNA+ATAC-seq experiments revealed ~11,000 cells. A-D) Quality control metrics reflecting chromatin accessibility (A, B) and transcriptomic (C, D) assays. Height indicates number of cells from each sample. *Blue=E13.5 palate, Yellow=E15.5 palate.* E) Initial UMAP projection labeled by initial cluster assignment per cell, from ArchR analysis of ~11,000 mouse palate cells from E13.5 and E15.5. F) UMAP projection as in (E), depicting the sample contributing each cells. G) UMAP projection as in (E, F) depicting highest gene ontology enrichment for each cluster, based on marker genes identified within that cluster. H) Annotated cell types based on GO enrichment from (G).

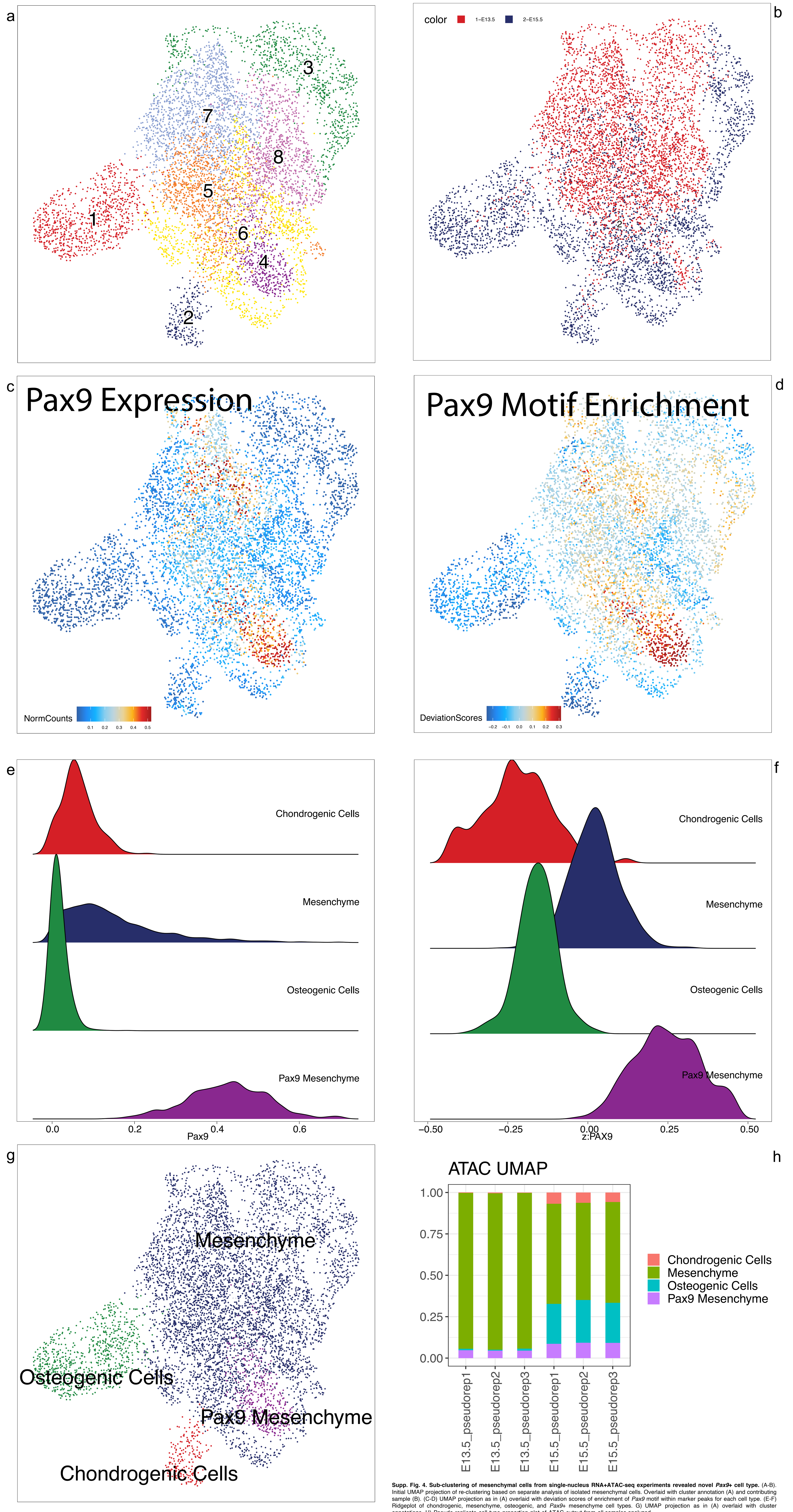

Supp. Fig. 4. Sub-clustering of mesenchymal cells from single-nucleus RNA+ATAC-seq experiments revealed novel *Pax9*+ cell type. (A-B). Initial UMAP projection of re-clustering based on separate analysis of isolated mesenchymal cells. Overlaid with cluster annotation (A) and contributing sample (B). (C-D) UMAP projection as in (A) overlaid with deviation scores of enrichment of *Pax9* motif within marker peaks for each cell type. (E-F) Ridgeplot of chondrogenic, mesenchyme, osteogenic, and *Pax9*+ mesenchyme cell types. G) UMAP projection as in (A) overlaid with cluster annotations. H) Pseudo-replicate cell type proportion plot of ATAC output from all samples analyzed.

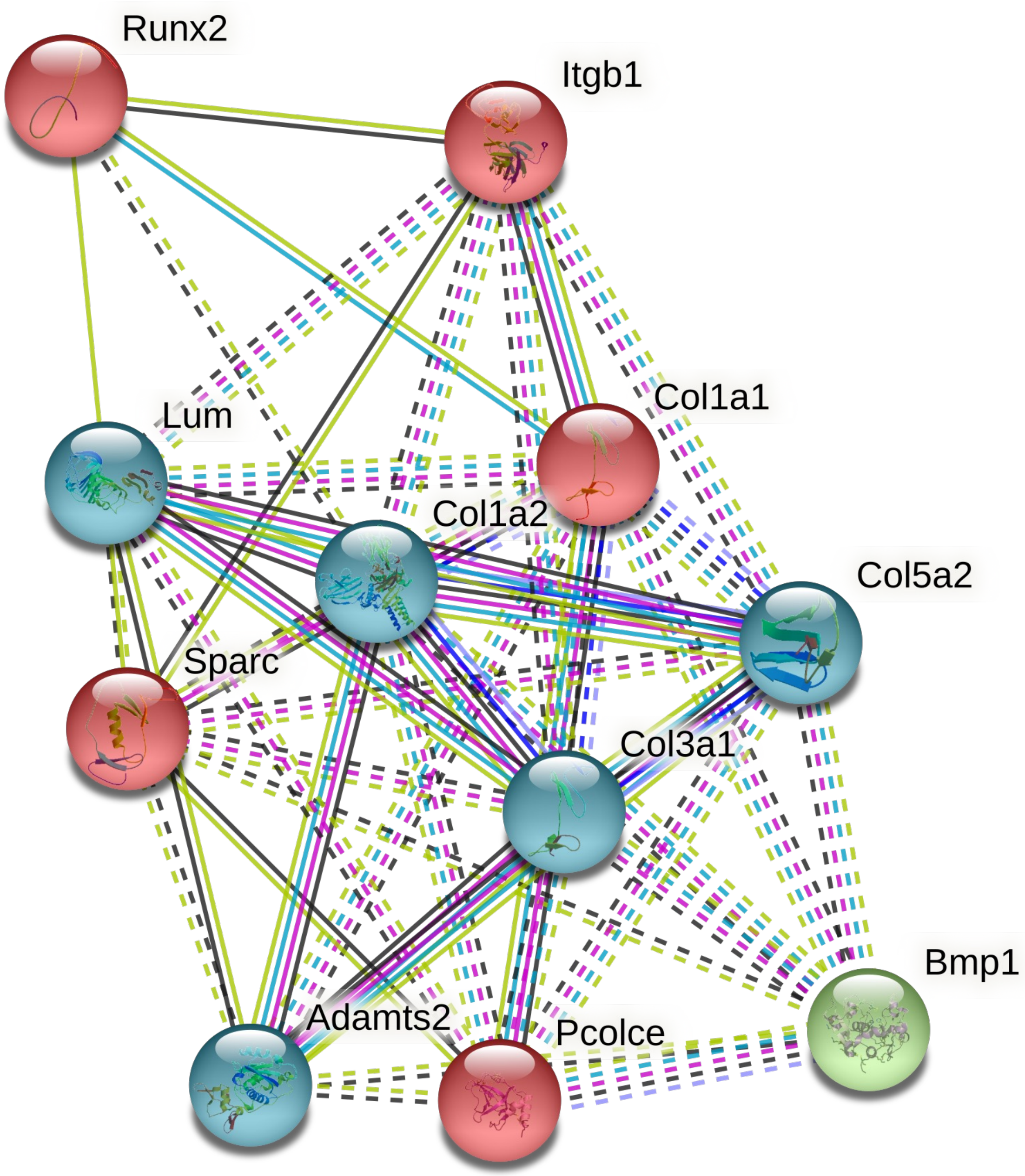

Cluster 1

Tissue Expression Database: **Osteoblast [BTO:0001593]**
(Col1a1, Sparc, Runx2)

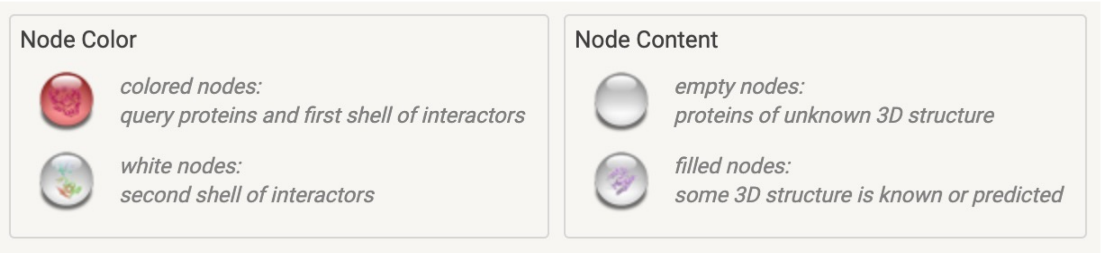

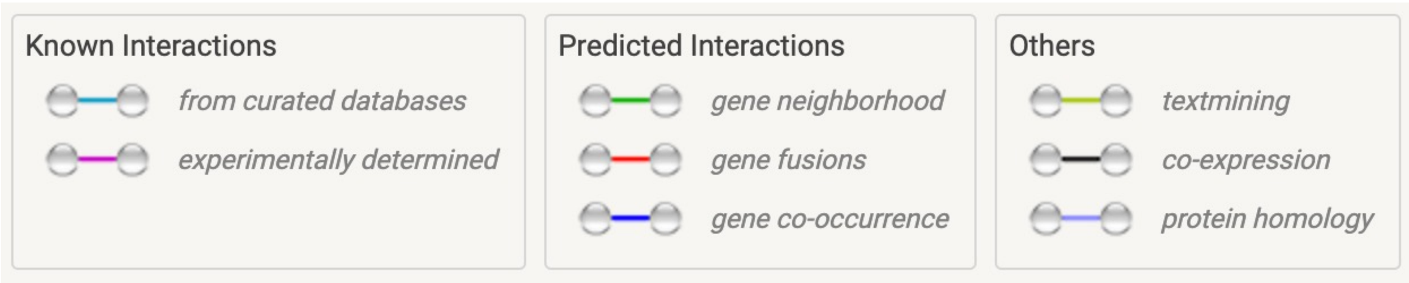

Supp. Fig. 5. From String Consortium Database, K-means clustering highlights osteoblast markers with common interactions with high strength of association (2.41) and low false discovery rate (1.20e-06).

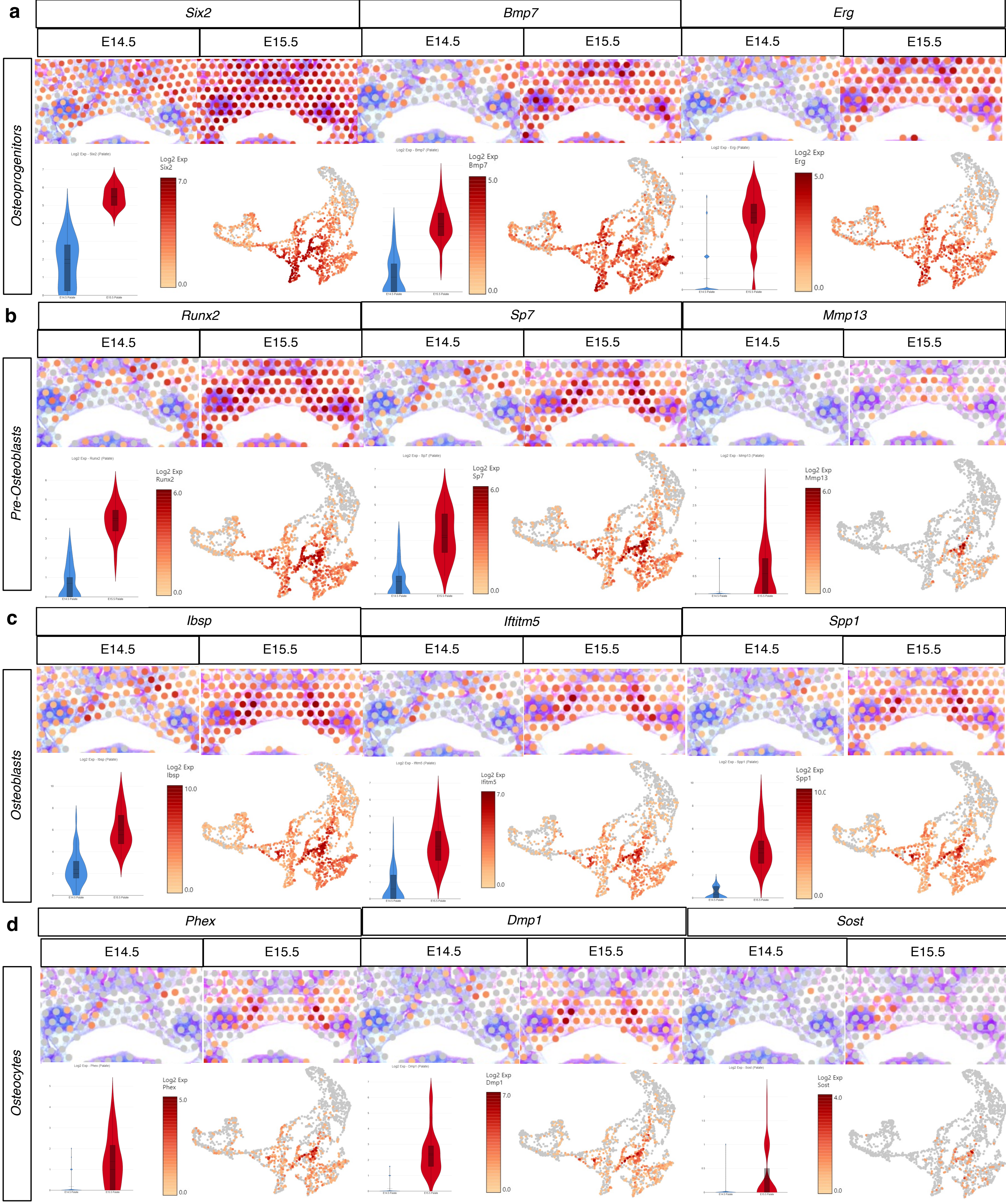

Supp. Fig. 6. ***In vivo* spRNAseq confirms temporal increase in osteogenic markers and provides spatial quantification of lineage-specific differentiation of condensed mesenchyme progenitors of palatal bone**. Markers of osteogenic lineage induction included the earliest **a** osteogenic progenitors *Six2, Erg*, and *Bmp7*, then **b** pre-osteoblast markers *Runx2*, *Sp7*, and *Mmp13*, followed by **c** more mature, functional osteoblast markers *Ifitm5, Ibsp*, and *Spp1*; finally, **d** markers of osteocyte activity *Sost, Dmp1*, and *Phex*, increase at this stage, yet to a lesser extent than earlier markers. Notably, markers from all early osteogenic cell types (osteoprogenitors, pre-osteoblasts, and osteoblasts) demonstrated some expressing at E14.5; however, all osteocyte conserved markers only appeared in substantial expression by E15.5.

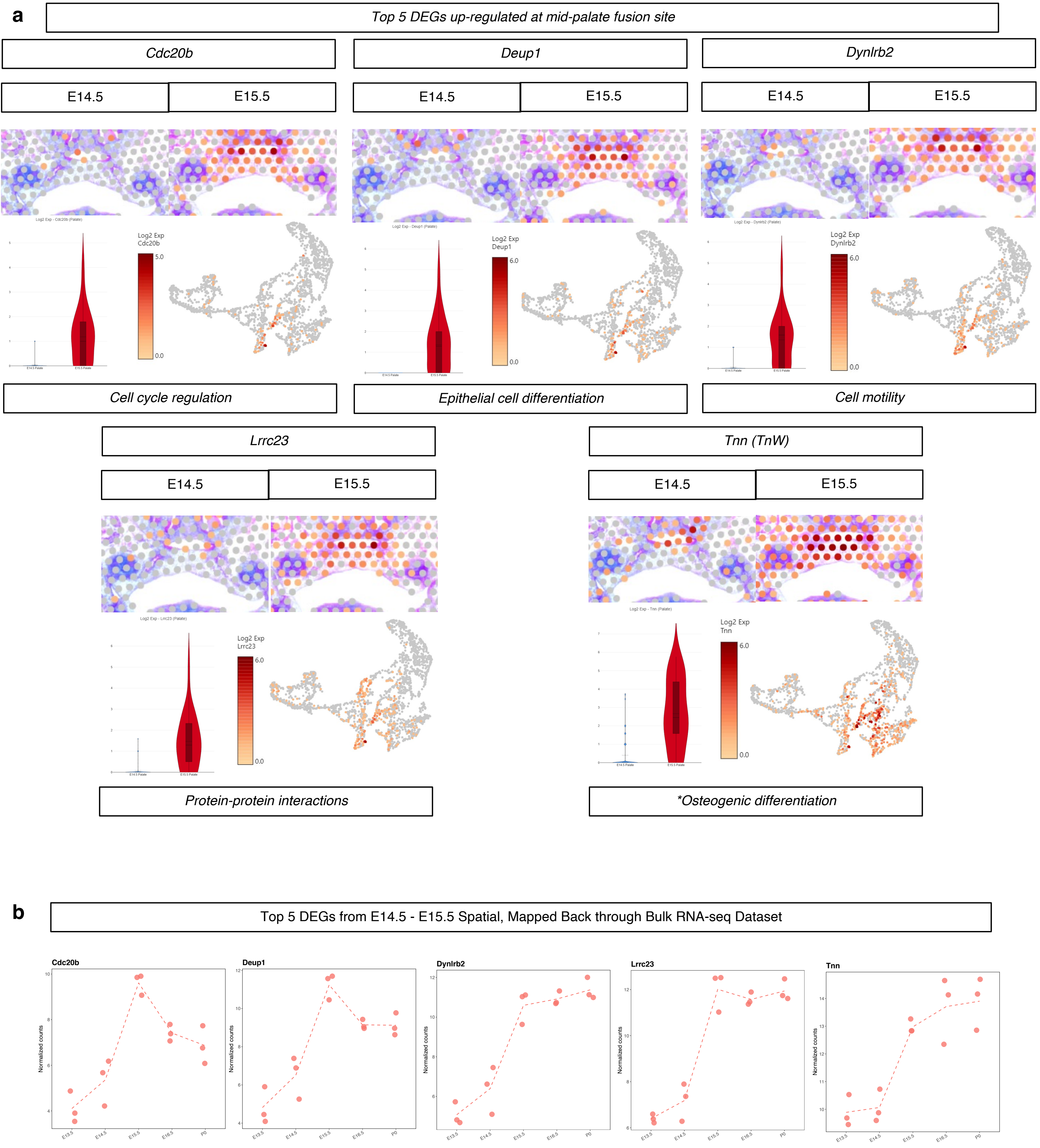

Supp. Fig. 7. Spatial RNA-seq-derived novel marker genes differentially expressed at the stage of palatal fusion. **a** Spatial *in vivo* expression, UMAPs, and violin plots from the top 5 marker genes from unbiased differential analysis of selective palate barcoding sequencing capture areas from E14.5 – E15.5 mid-palate coronal sections. **b** Gene plots from previously curated bulk RNA-seq analyses corroborate above spRNA-seq data, further evidence of the potential important functional roles for *Cdc20b*, *Deup1*, *Dynlrb2*, *Lrrc23*, and *Tnn* (aka *Tn-W*) in secondary palate development.
