# Supplementary material for: Multimodal spatiotemporal transcriptomic resolution of embryonic palate osteogenesis
Source: Nat Commun. 2023 Sep 14;14:5687. doi: 10.1038/s41467-023-41349-9 (PMC10502152; doi:10.1038/s41467-023-41349-9)
Supplement: Supplementary file 2 — Reporting Summary [file 41467_2023_41349_MOESM2_ESM.pdf]

nature portfolio

Corresponding author(s): Jeremie Oliver Piña NCOMMS-22-37523D

Last updated by author(s): Aug 22, 2023

Reporting Summary

Nature Portfolio wishes to improve the reproducibility of the work that we publish. This form provides structure for consistency and transparency in reporting. For further information on Nature Portfolio policies, see our Editorial Policies and the Editorial Policy Checklist.

Statistics

For all statistical analyses, confirm that the following items are present in the figure legend, table legend, main text, or Methods section.

n/a	Confirmed	
☐	☒	The exact sample size (*n*) for each experimental group/condition, given as a discrete number and unit of measurement
☐	☒	A statement on whether measurements were taken from distinct samples or whether the same sample was measured repeatedly
☐	☒	The statistical test(s) used AND whether they are one- or two-sided<br>*Only common tests should be described solely by name; describe more complex techniques in the Methods section.*
☐	☒	A description of all covariates tested
☐	☒	A description of any assumptions or corrections, such as tests of normality and adjustment for multiple comparisons
☐	☒	A full description of the statistical parameters including central tendency (e.g. means) or other basic estimates (e.g. regression coefficient) AND variation (e.g. standard deviation) or associated estimates of uncertainty (e.g. confidence intervals)
☐	☒	For null hypothesis testing, the test statistic (e.g. *F*, *t*, *r*) with confidence intervals, effect sizes, degrees of freedom and *P* value noted<br>*Give P values as exact values whenever suitable.*
☒	☐	For Bayesian analysis, information on the choice of priors and Markov chain Monte Carlo settings
☐	☒	For hierarchical and complex designs, identification of the appropriate level for tests and full reporting of outcomes
☒	☐	Estimates of effect sizes (e.g. Cohen's *d*, Pearson's *r*), indicating how they were calculated

Our web collection on statistics for biologists contains articles on many of the points above.

Software and code

Policy information about availability of computer code

Data collection	For each stage of analysis, 3 technical replicates and 3 biological replicates were included for sequencing. 1-4 ug of total RNA samples were purified with PolyA extraction, and then purified mRNAs were constructed to RNA-Seq libraries with specific barcodes using illumina TruSeq Stranded mRNA Library Prep Kit. All the RNA-Seq libraries were pooled together and sequenced using illumina NovaSeq to generate approximately ~40 million 2x100 paired-end reads for each sample. The raw data were demultiplexed and analyzed further. Raw sequence reads were processed using lcdb-wf v1.9rc (lcdb.github.io/lcdb-wf/) according to the following steps: Raw sequence reads were trimmed with cutadapt v3.4 to remove any adapters while performing light quality trimming with parameters '-a AGATCGGAAGAGCACACGTCTGAACTCCAGTCA -A AGATCGGAAGAGCGTCGTGTAGGGAAAGAGTGT -q 20 –minimum-length = 25.'
Data analysis	Patterns of expression profile were computed and plotted using the function degPatterns from DEGreport v1.30.0 (R package version 1.30.3, http://lpantano.github.io/DEGreport/). Functional enrichment was performed for GO Biological Process, Cellular Component and Molecular Function using the ClusterProfiler v 4.2.0 function go.enrich. Detailed scripts of single-nucleus RNA-seq analysis can be found on the Cotney Lab GitHub (https://github.com/emmawwinchester/mousepalate) and detailed methodology for bioinformatic analysis in the Methods section.

For manuscripts utilizing custom algorithms or software that are central to the research but not yet described in published literature, software must be made available to editors and reviewers. We strongly encourage code deposition in a community repository (e.g. GitHub). See the Nature Portfolio guidelines for submitting code & software for further information.

Data

Policy information about availability of data

All manuscripts must include a data availability statement. This statement should provide the following information, where applicable:

- Accession codes, unique identifiers, or web links for publicly available datasets
- A description of any restrictions on data availability
- For clinical datasets or third party data, please ensure that the statement adheres to our policy

All results and analysis data are available in the main text or the supplementary materials. Additional information and materials are available from the corresponding author upon reasonable request. All sequencing data reported in this paper are deposited in GEO under the SuperSeries GSE205449.

Human research participants

Policy information about studies involving human research participants and Sex and Gender in Research.

Reporting on sex and gender	N/A
Population characteristics	N/A
Recruitment	N/A
Ethics oversight	N/A

Note that full information on the approval of the study protocol must also be provided in the manuscript.

Field-specific reporting

Please select the one below that is the best fit for your research. If you are not sure, read the appropriate sections before making your selection.

- [x] Life sciences
- [ ] Behavioural & social sciences
- [ ] Ecological, evolutionary & environmental sciences

For a reference copy of the document with all sections, see nature.com/documents/nr-reporting-summary-flat.pdf

Life sciences study design

All studies must disclose on these points even when the disclosure is negative.

Sample size	For each stage of analysis, 3 technical replicates and 3 biological replicates were included for sequencing, as described above. All in vivo validation experiments were performed in biological triplicate.
Data exclusions	No data were excluded
Replication	The use of technical and biological triplicate samples in the RNA-seq analysis allowed for verification of reproducibility of sequencing. Similarly, all quality control metrics were reported and validated at each step of the sequencing studies.
Randomization	All samples used in this study were of the same C57B6/J wild type background, thus, no randomization was necessary.
Blinding	Similar to above, no blinding was necessary given the uniform genetic background of each sample studied.

Reporting for specific materials, systems and methods

We require information from authors about some types of materials, experimental systems and methods used in many studies. Here, indicate whether each material, system or method listed is relevant to your study. If you are not sure if a list item applies to your research, read the appropriate section before selecting a response.

Materials & experimental systems

n/a	Involved in the study
☒	☐ Antibodies
☒	☐ Eukaryotic cell lines
☒	☐ Palaeontology and archaeology
☐	☒ Animals and other organisms
☒	☐ Clinical data
☒	☐ Dual use research of concern

Methods

n/a	Involved in the study
☒	☐ ChIP-seq
☒	☐ Flow cytometry
☒	☐ MRI-based neuroimaging

Animals and other research organisms

Policy information about studies involving animals; ARRIVE guidelines recommended for reporting animal research, and Sex and Gender in Research

Laboratory animals	All samples used in this study were of the same C57B6/J wild type background. C57BL/6J mice were obtained from the Jackson Laboratory. Inbred strain of female C57BL/6 mice were utilized for all experiments. Healthy fertile male mice were mated with the same strain C57BL/6J female mice during sexual maturity (6-12 weeks of age). Timed pregnancies were conducted via vaginal plug identification, with day 0.5 indicating date of identification.
Wild animals	N/A
Reporting on sex	RNA samples collected from embryos used for analysis in this study included both female and male sex at each stage of analysis confirmed by marker gene profiling for sex-specific genes during development. No differential analyses were conducted between male vs female biological samples in these studies, as all pooled samples were mixed male and female sexes.
Field-collected samples	N/A
Ethics oversight	All animal procedures were approved by the National Institutes of Health, National Institute of Child Health and Human Development Animal Care and Use Committee (ACUC), under Animal Study Protocol (ASP) #21-031.

Note that full information on the approval of the study protocol must also be provided in the manuscript.
